# Supplementary material for: Does One Size Fit All? Variations in the DNA Barcode Gaps of Macrofungal Genera
Source: J Fungi (Basel). 2023 Jul 26;9(8):788. doi: 10.3390/jof9080788 (PMC10455624; doi:10.3390/jof9080788)
Supplement: Supplementary file 1 [file jof-09-00788-s001.zip › jof-2422183-supplementary.pdf]

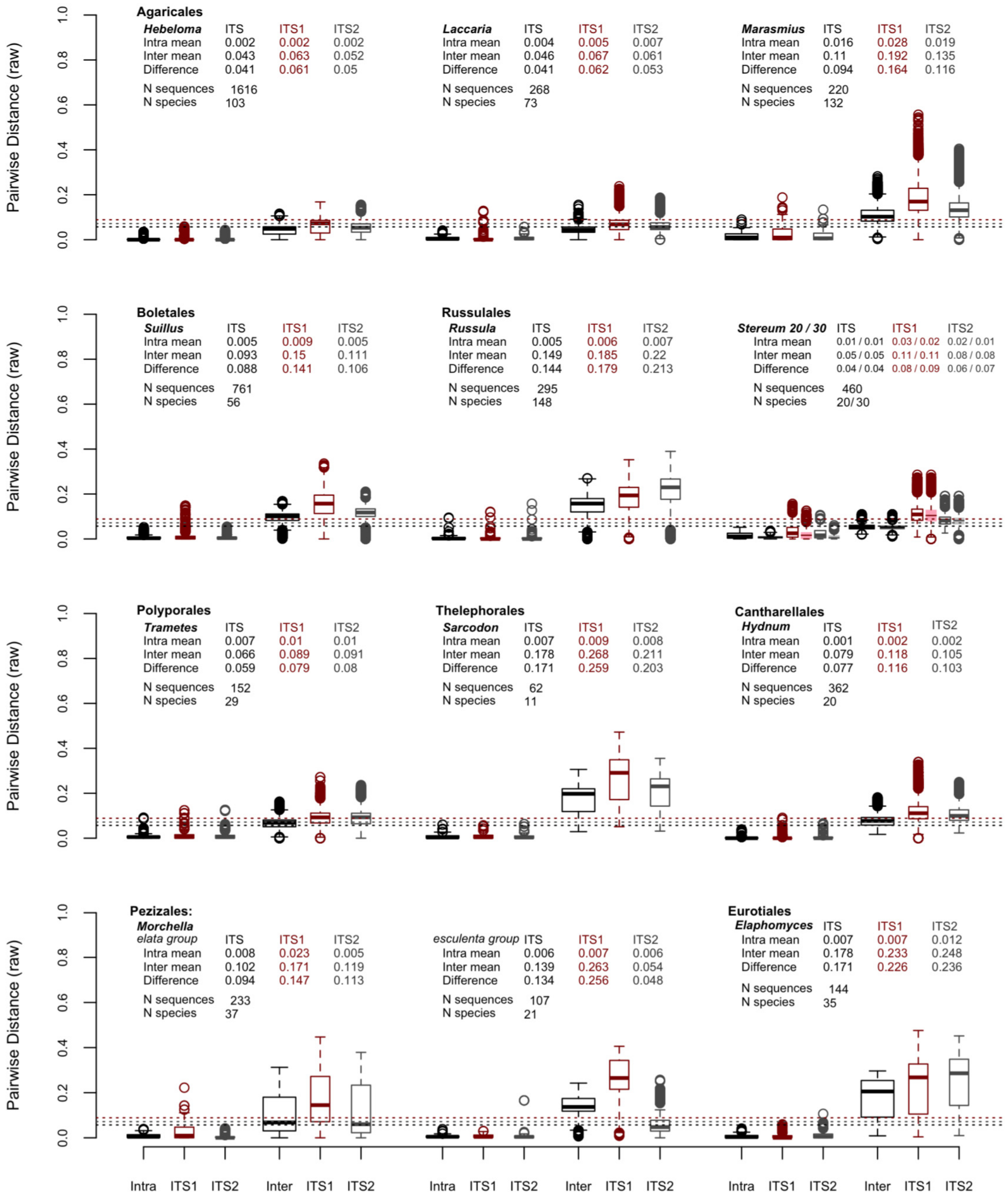

Figure S1 Pairwise distances of DNA barcode sequence data using a raw substitution model. Horizontal lines are the inter-specific means for nrITS, ITS1 and ITS2 pairwise distance distributions from 5146 sequences. *Stereum* is evaluated under two partitions. The “lumping” partition has 20 species and is represented by open boxplots. The “splitting” partition has 30 species and is represented in the filled boxplots.

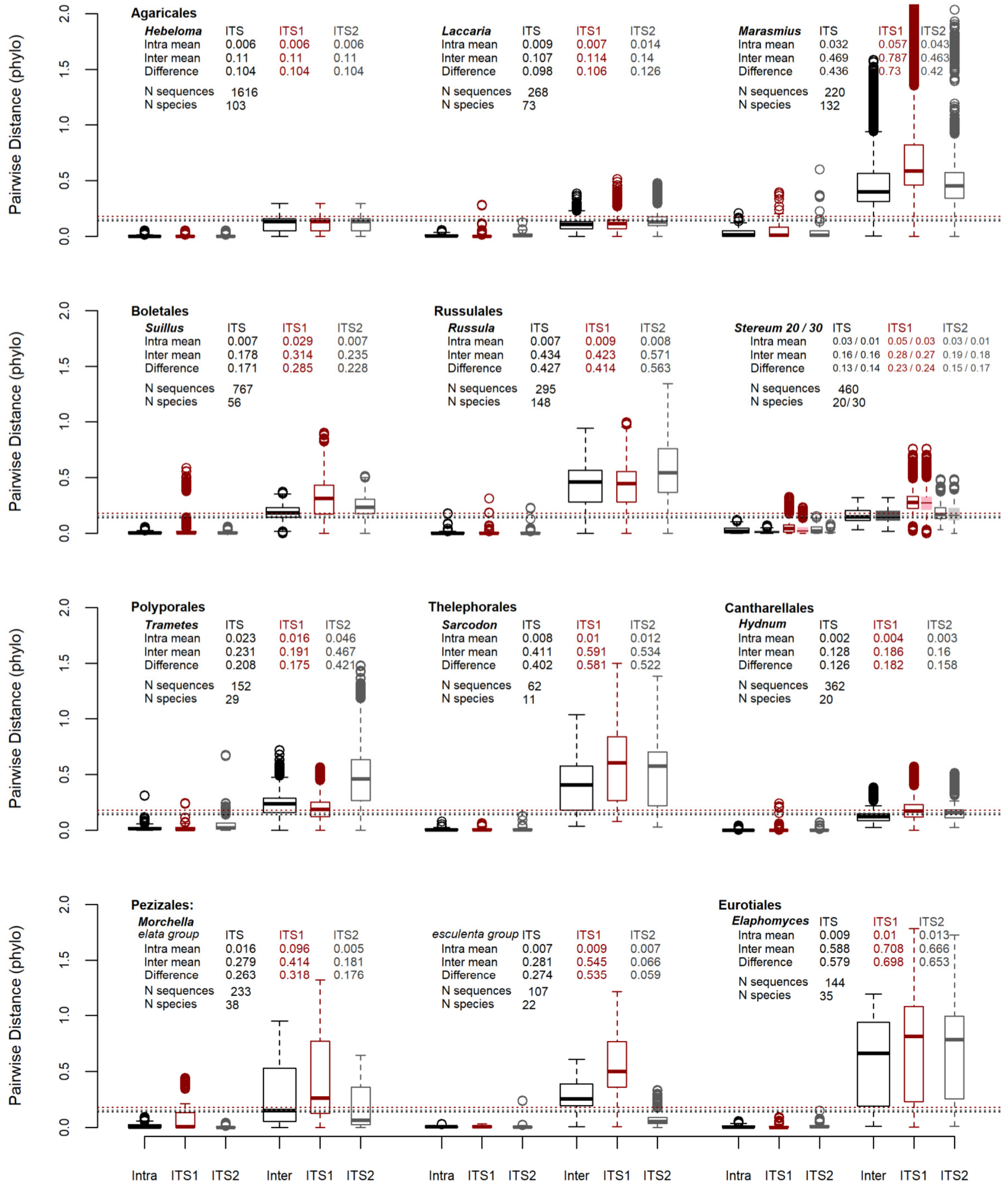

Figure S2 Pairwise distances of DNA barcode sequence data using phylogenetic pairwise distances. Horizontal lines are the inter-specific means for nrITS, ITS1 and ITS2 pairwise distance distributions from 5146 sequences. *Stereum* is evaluated under two partitions. The "lumping" partition has 20 species and is represented by open boxplots. The "splitting" partition has 30 species and is represented in the filled boxplots.

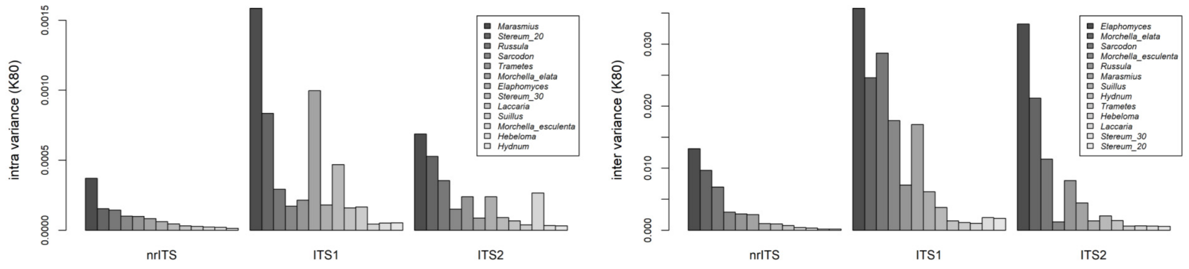

Figure S3 - Variances in pairwise distance distributions (K80 model) ranked nrITS sequence data.

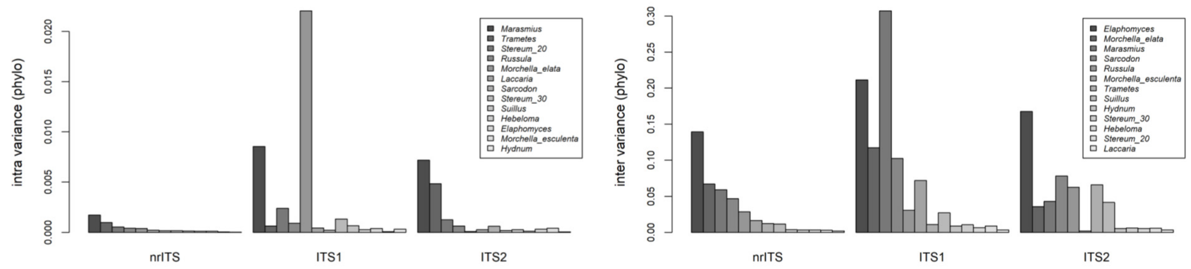

Figure S4 - Variances in phylogenetic pairwise distances ranked nrITS sequence data.

Table S1 Bar Code Gap Assessment of DNA sequence data from Macrofungal Genera (raw model data)

			**85% Quantiles**						**90% Quantiles**						**95% Quantiles**					
			**nrITS**		**ITS1**		**ITS2**		**nrITS**		**ITS1**		**ITS2**		**nrITS**		**ITS1**		**ITS2**	
**Name**	**Ntaxa**	**Nseq**	**gap?**	**size**	**gap?**	**size**	**gap?**	**size**	**gap?**	**size**	**gap?**	**size**	**gap?**	**size**	**gap?**	**size**	**gap?**	**size**	**gap?**	**size**
Hebeloma	103	1616	**TRUE**	0.0051	**TRUE**	0.0042	**TRUE**	0.0051	**TRUE**	0.0016	FALSE		**TRUE**	0.0000	FALSE		FALSE		FALSE	
Laccaria	73	268	**TRUE**	0.0117	**TRUE**	0.0090	**TRUE**	0.0100	**TRUE**	0.0061	FALSE		**TRUE**	0.0097	**TRUE**	0.0017	FALSE		**TRUE**	0.0000
Marasmius	132	220	**TRUE**	0.0141	**TRUE**	0.0106	**TRUE**	0.0040	**TRUE**	0.0072	FALSE		FALSE		FALSE		FALSE		FALSE	
Suillus	56	767	**TRUE**	0.0221	**TRUE**	0.0145	**TRUE**	0.0316	**TRUE**	0.0138	FALSE		**TRUE**	0.0225	**TRUE**	0.0012	FALSE		**TRUE**	0.0038
Russula	148	295	**TRUE**	0.0708	**TRUE**	0.0652	**TRUE**	0.1045	**TRUE**	0.0600	**TRUE**	0.0484	**TRUE**	0.0798	**TRUE**	0.0355	**TRUE**	0.0253	**TRUE**	0.0469
"lumping"	20	460	**TRUE**	0.0035	FALSE		FALSE	-0.0032	FALSE		FALSE		FALSE		FALSE		FALSE			
"splitting"	30	460	**TRUE**	0.0151	FALSE		**TRUE**	0.0306	**TRUE**	0.0110	FALSE		**TRUE**	0.0219	**TRUE**	0.0055	FALSE		**TRUE**	0.0119
Trametes	29	152	**TRUE**	0.0720	**TRUE**	0.1011	**TRUE**	0.1007	**TRUE**	0.0739	**TRUE**	0.1058	**TRUE**	0.1048	**TRUE**	0.0927	**TRUE**	0.1243	**TRUE**	0.1175
Sarcodon	11	62	**TRUE**	0.0386	**TRUE**	0.0423	**TRUE**	0.0479	**TRUE**	0.0145	**TRUE**	0.0389	**TRUE**	0.0131	FALSE		**TRUE**	0.0252	**TRUE**	0.0001
Hydnum	20	362	**TRUE**	0.0408	**TRUE**	0.0521	**TRUE**	0.0475	**TRUE**	0.0336	**TRUE**	0.0399	**TRUE**	0.0424	**TRUE**	0.0258	**TRUE**	0.0245	**TRUE**	0.0297
elata group	37	233	FALSE		FALSE		FALSE		FALSE		FALSE		FALSE		FALSE		FALSE		FALSE	
esculenta group	21	107	**TRUE**	0.0293	**TRUE**	0.0567	**TRUE**	0.0042	**TRUE**	0.0190	**TRUE**	0.0385	FALSE		**TRUE**	0.0028	**TRUE**	0.0079	FALSE	
Elaphomyces	35	144	**TRUE**	0.0373	**TRUE**	0.0354	**TRUE**	0.0350	**TRUE**	0.0099	FALSE		**TRUE**	0.0184	FALSE		FALSE		FALSE	
			**Count**	**AVE**	**Count**	**AVE**	**Count**	**AVE**	**Count**	**AVE**	**Count**	**AVE**	**Count**	**AVE**	**Count**	**AVE**	**Count**	**AVE**	**Count**	**AVE**
			12	0.0300	10	0.0391	11	0.0348	11	0.0228	5	0.0543	9	0.0347	7	0.0236	5	0.0414	7	0.0300

Table S2 Bar Code Gap Assessment of DNA sequence data from Macrofungal Genera (phylo model data)

			**85% Quantiles**						**90% Quantiles**						**95% Quantiles**					
			**nrITS**		**ITS1**		**ITS2**		**nrITS**		**ITS1**		**ITS2**		**nrITS**		**ITS1**		**ITS2**	
**Name**	**Ntaxa**	**Nseq**	**gap?**	**size**	**gap?**	**size**	**gap?**	**size**	**gap?**	**size**	**gap?**	**size**	**gap?**	**size**	**gap?**	**size**	**gap?**	**size**	**gap?**	**size**
Hebeloma	103	1616	FALSE		FALSE		**TRUE**	0.0005	FALSE		FALSE		FALSE		FALSE		FALSE		FALSE	
Laccaria	73	268	**TRUE**	0.0427	**TRUE**	0.0370	**TRUE**	0.0505	**TRUE**	0.0316	**TRUE**	0.0256	**TRUE**	0.0377	**TRUE**	0.0134	FALSE		**TRUE**	0.0156
Marasmius	132	220	**TRUE**	0.2069	**TRUE**	0.2849	**TRUE**	0.2011	**TRUE**	0.1613	**TRUE**	0.2314	**TRUE**	0.1154	**TRUE**	0.0593	FALSE		**TRUE**	0.0003
Suillus	56	767	**TRUE**	0.1388	**TRUE**	0.0902	**TRUE**	0.2587	**TRUE**	0.0792	**TRUE**	0.0422	**TRUE**	0.1509	**TRUE**	0.0208	FALSE		**TRUE**	0.0562
Russula	148	295	**TRUE**	0.2200	**TRUE**	0.1968	**TRUE**	0.2906	**TRUE**	0.1856	**TRUE**	0.1556	**TRUE**	0.2402	**TRUE**	0.1300	**TRUE**	0.0961	**TRUE**	0.1497
Stereum																				
"lumping"	20	460	**TRUE**	0.0524	**TRUE**	0.0828	**TRUE**	0.0159	**TRUE**	0.0430	**TRUE**	0.0327	**TRUE**	0.0012	**TRUE**	0.0231	FALSE		FALSE	
"splitting"	30	460	**TRUE**	0.0735	**TRUE**	0.0835	**TRUE**	0.0691	**TRUE**	0.0523	**TRUE**	0.0493	**TRUE**	0.0606	**TRUE**	0.0207	FALSE		**TRUE**	0.0438
Trametes	29	152	**TRUE**	0.0723	**TRUE**	0.0478	**TRUE**	0.1042	**TRUE**	0.0252	**TRUE**	0.0058	**TRUE**	0.0497	FALSE		FALSE		FALSE	
Sarcodon	11	62	**TRUE**	0.1472	**TRUE**	0.2078	**TRUE**	0.1754	**TRUE**	0.0639	**TRUE**	0.0733	**TRUE**	0.0735	**TRUE**	0.0109	**TRUE**	0.0582	**TRUE**	0.0019
Hydnum	20	362	**TRUE**	0.0722	**TRUE**	0.0892	**TRUE**	0.0961	**TRUE**	0.0656	**TRUE**	0.0763	**TRUE**	0.0855	**TRUE**	0.0443	**TRUE**	0.0541	**TRUE**	0.0587
Morchella																				
elata group	37	233	FALSE		FALSE		**TRUE**	0.0037	FALSE		FALSE		FALSE		FALSE		FALSE		FALSE	
esculenta group	21	107	**TRUE**	0.1731	**TRUE**	0.3104	**TRUE**	0.0170	**TRUE**	0.1595	**TRUE**	0.2635	FALSE	0.0088	**TRUE**	0.0250	**TRUE**	0.0464	FALSE	
Elaphomyces	35	144	**TRUE**	0.1298	**TRUE**	0.1536	**TRUE**	0.1598	**TRUE**	0.0838	**TRUE**	0.1224	**TRUE**	0.0810	**TRUE**	0.0233	**TRUE**	0.0015	**TRUE**	0.0265
			**Count**	**AVE**	**Count**	**AVE**	**Count**	**AVE**	**Count**	**AVE**	**Count**	**AVE**	**Count**	**AVE**	**Count**	**AVE**	**Count**	**AVE**	**Count**	**AVE**
			11	0.1208	11	0.1440	13	0.1110	11	0.0864	11	0.0980	10	0.0822	10	0.0371	5	0.0513	8	0.0441

Table S3 - Means and Variances from Pairwise Distances model K80

			nrITS				ITS1				ITS2			
			intra		inter		intra		inter		intra		inter	
**Genus**	**N_seq**	**N_sp**	**Mean**	**Variance**	**Mean**	**Variance**	**Mean**	**Variance**	**Mean**	**Variance**	**Mean**	**Variance**	**Mean**	**Variance**
Hebeloma	1616	103	0.0015757	2.06E-05	0.0445008	0.0004389	0.0021881	5.09E-05	0.0674602	0.0012204	0.0020833	3.24E-05	0.0543934	0.0006689
Laccaria	268	73	0.0044947	3.17E-05	0.0474823	0.0003309	0.0052429	0.00016	0.07193	0.001118	0.007473	6.63E-05	0.0644148	0.0006933
Marasmius	220	132	0.0165397	0.0003718	0.1215275	0.0025093	0.0299236	0.001585	0.2345916	0.0170349	0.0196264	0.0006877	0.1531523	0.0044087
Suillus	767	56	0.0052758	2.87E-05	0.1002512	0.0010661	0.0089584	0.0001681	0.1726107	0.0061791	0.0052632	3.90E-05	0.1227168	0.001528
Russula	295	148	0.0050125	0.0001431	0.1710109	0.0026277	0.0065639	0.0002923	0.2242155	0.0072993	0.0068533	0.0003547	0.2725789	0.0080198
Stereum_20	460	20	0.0147434	0.0001545	0.055838	0.0001749	0.0323228	0.0008361	0.1210011	0.0019126	0.0221083	0.0005271	0.0893946	0.0006254
Stereum_30	460	30	0.0078066	4.52E-05	0.054236	0.000204	0.0196841	0.0004686	0.1172022	0.0020325	0.0090274	9.03E-05	0.0870243	0.0006829
Trametes	152	29	0.0074422	9.93E-05	0.0660843	0.0007638	0.0103076	0.0002141	0.089289	0.0015113	0.0100698	0.0002393	0.0905635	0.0015294
Sarcodon	62	11	0.0068615	0.0001015	0.2109419	0.006972	0.0092146	0.0001716	0.3591266	0.0285637	0.0077835	0.0001507	0.2626914	0.0114783
Hydnum	362	20	0.0014628	1.28E-05	0.0851077	0.0010421	0.0020239	5.28E-05	0.1327558	0.0036654	0.0021187	3.08E-05	0.1180398	0.0023055
Morchella_elata	233	37	0.0083597	8.26E-05	0.1162583	0.0096621	0.0246072	0.0009969	0.2103976	0.0245806	0.0052264	8.70E-05	0.1434072	0.0212878
Morchella_esculenta	107	21	0.0055933	2.20E-05	0.1567747	0.0029188	0.007131	4.38E-05	0.3401207	0.0176755	0.0065769	0.000266	0.0574243	0.001353
Elaphomyces	144	35	0.0068132	6.15E-05	0.2156777	0.0131417	0.0072871	0.0001816	0.3109116	0.0357568	0.0119193	0.0002387	0.3339908	0.0332489
		Averages	0.0070755	9.041E-05	0.111207	0.0032194	0.0127273	0.0004017	0.1885856	0.0114269	0.008933	0.0002162	0.1422917	0.0067562

Table S4 - Means and Variances from Pairwise Distances model raw

			nrITS				ITS1				ITS2			
			intra		inter		intra		inter		intra		inter	
Genus	N_seq	N_sp	Mean	Variance	Mean	Variance	Mean	Variance	Mean	Variance	Mean	Variance	Mean	Variance
Hebeloma	1616	103	0.04266	1.97E-05	0.001556	0.000387	0.063158	4.73E-05	0.002139	0.001011	0.002054	3.10E-05	0.051611	0.000564
Laccaria	268	73	0.045576	3.06E-05	0.004457	0.000282	0.067472	0.000143	0.005112	0.000875	0.007383	6.40E-05	0.060754	0.000546
Marasmius	220	132	0.110148	0.000342	0.016079	0.001708	0.191746	0.001316	0.028203	0.007549	0.018868	0.000612	0.134685	0.002611
Suillus	767	56	0.092737	2.71E-05	0.005183	0.00084	0.149962	0.000151	0.008695	0.003962	0.005146	3.67E-05	0.111193	0.001113
Russula	295	148	0.149145	0.000126	0.004884	0.001664	0.185164	0.000251	0.006312	0.003766	0.006548	0.000288	0.219921	0.00383
Stereum_20	460	20	0.053463	0.000146	0.014455	0.000146	0.109323	0.000726	0.030769	0.001266	0.021372	0.000481	0.083314	0.000471
Stereum_30	460	30	0.051964	4.37E-05	0.00772	0.000172	0.106038	0.000421	0.019025	0.001368	0.008878	8.62E-05	0.081198	0.000519
Trametes	152	29	0.066084	9.93E-05	0.007442	0.000764	0.089289	0.000214	0.010308	0.001511	0.01007	0.000239	0.090563	0.001529
Sarcodon	62	11	0.177566	9.55E-05	0.006752	0.004111	0.268318	0.000161	0.009009	0.011294	0.007627	0.000141	0.210678	0.005689
Hydnum	362	20	0.078703	1.23E-05	0.001451	0.000752	0.117832	4.77E-05	0.001976	0.002301	0.00209	2.90E-05	0.105162	0.001418
Morchella_elata	233	37	0.102045	7.96E-05	0.008254	0.006473	0.17099	0.000866	0.023496	0.012616	0.005145	8.32E-05	0.118543	0.012531
Morchella_esculenta	107	21	0.139117	2.13E-05	0.005554	0.002003	0.263222	4.26E-05	0.00706	0.007771	0.006375	0.000212	0.054096	0.001067
Elaphomyces	144	35	0.177519	5.94E-05	0.006729	0.007367	0.232922	0.00017	0.007111	0.014911	0.011624	0.00022	0.247963	0.01304
		Averages	0.098979	8.48E-05	0.006963	0.002051	0.155033	0.000351	0.012247	0.0054	0.008706	0.000194	0.120745	0.003456

Table S5 - Means and Variances from Phylogenetic Pairwise Distances

			nrITS				ITS1				ITS2			
			intra		inter		intra		inter		intra		inter	
**Genus**	**N_seq**	**N_sp**	**Mean**	**Variance**	**Mean**	**Variance**	**Mean**	**Variance**	**Mean**	**Variance**	**Mean**	**Variance**	**Mean**	**Variance**
Hebeloma	1616	103	0.005999	0.000113	0.109538	0.00333	0.010234	0.00026	0.143781	0.006455	0.00672	0.000123	0.130488	0.005438
Laccaria	268	73	0.008977	0.000225	0.106578	0.001851	0.007227	0.00043	0.113649	0.00314	0.014286	0.00025	0.140257	0.003419
Marasmius	220	132	0.032491	0.001719	0.46864	0.059019	0.056896	0.008556	0.786982	0.30715	0.043472	0.007186	0.463358	0.04292
Suillus	767	56	0.010571	0.000132	0.287703	0.011649	0.01433	0.000667	0.332606	0.0272	0.013816	0.000256	0.495996	0.041412
Russula	295	148	0.007417	0.000403	0.434074	0.028511	0.009313	0.000909	0.423181	0.030653	0.008238	0.000638	0.571401	0.062741
Stereum_20	460	20	0.026836	0.000531	0.16179	0.002829	0.05308	0.002399	0.281428	0.008859	0.032725	0.001249	0.185093	0.00556
Stereum_30	460	30	0.014282	0.000164	0.155029	0.003331	0.034119	0.001325	0.269653	0.010467	0.012407	0.00018	0.17836	0.005908
Trametes	152	29	0.022992	0.000973	0.230835	0.012386	0.015859	0.000634	0.190978	0.01089	0.045827	0.00485	0.466954	0.066127
Sarcodon	62	11	0.00836	0.000167	0.410664	0.04664	0.010446	0.00021	0.591415	0.10274	0.011764	0.000619	0.533756	0.07817
Hydnum	362	20	0.001827	1.78E-05	0.127548	0.003673	0.003527	0.0003	0.185697	0.008816	0.002537	3.91E-05	0.160137	0.005225
Morchella_elata	233	37	0.015652	0.000391	0.279088	0.067153	0.096207	0.022043	0.414101	0.117487	0.005448	9.27E-05	0.181127	0.035877
Morchella_esculenta	107	21	0.007157	2.88E-05	0.28102	0.016438	0.009154	6.17E-05	0.544592	0.072025	0.007003	0.000421	0.065803	0.001868
Elaphomyces	144	35	0.008871	0.000107	0.587917	0.139614	0.009976	0.000374	0.707646	0.21163	0.013258	0.000301	0.666306	0.167879
		Averages	0.013187	0.000382	0.280033	0.030494	0.025413	0.002936	0.383516	0.070578	0.016731	0.001247	0.32608	0.040196
